# Child-to-Parent Violence and Abuse: A Scoping Review

**DOI:** 10.1177/15248380241246033

**Published:** 2024-04-29

**Authors:** Michaela M. Rogers, Charlotte Ashworth

**Affiliations:** 1 The University of Sheffield, UK

**Keywords:** adolescent-to-parent violence, adolescent-to-parent abuse, childhood aggression, domestic abuse, family violence, parent abuse, parricide

## Abstract

Child-to-parent violence and abuse (CPVA) is a pattern of behavior where a parent or carer is abused by a child they are caring for. The main body of work on CPVA is relatively recent and evolving at pace. This scoping review explores the characteristics of parents, carers, children, and young people in cases of CPVA, the characteristics of CPVA, and barriers to and facilitators of help-seeking in cases of CPVA. The scoping review did not exclude any studies on the basis of geographical location or date of the study. The databases Scopus, CINAHL, Web of Science, Medline, and PubMed were searched in August 2023, along with hand searches of key journals. A total of 145 reports were included in the review, selected for their relevance to the scoping review questions. The main findings were: (a) the field of CPVA is rapidly growing, doubling in the last decade but with a predominance of quantitative studies; (b) there is no agreed universal definition; (c) children and young people with disabilities; who identify as trans or nonbinary gender, or who are adopted or fostered, are almost completely absent from the existing research; (d) there is very limited research focusing on protective factors or on help-seeking.

## Introduction

Child-to-parent violence and abuse (CPVA) was first identified as “battered parent syndrome” (Harbin & Madden, 1979), and there are a number of different definitions of this phenomenon in the research literature (Cottrell, 2004; Holt, 2013; Wilcox, 2012). Some of these terms, such as “adolescent-to-parent abuse”, exclude families with younger children experiencing CPVA. For the purpose of this scoping review, the term CPVA is adopted as inclusive of children aged 0 to 18 years old and their families.

A frequently referenced definition of CPVA describes it as “a pattern of behavior that uses verbal, financial, physical or emotional means to practice power and exert control over a parent” (Holt, 2013, p. 2). This definition is particularly helpful as it draws attention to the centrality of power and control in CPVA and the impact this may have on parent and child interactions. However, not all CPVA research asserts that violence is a form of power and control. In terms of impacts, CPVA can cause short-term and long-term harm to parents and carers, children and young people, and the wider family. Harms commonly reported include feelings of fear and stress, physical and mental health concerns, work and financial difficulties, social isolation, and problems in wider social and family relationships (Cottrell & Monk, 2004; Jackson, 2003; Holt, 2013; Holt, 2016a, 2016b).

In this review, we operationalize “child” to refer to legal status in which a child is aged under 18 (although some studies include a higher upper age limit), and “parent” refers to a biological or legal parent or another carer of a child, whether this is a family member or foster carer. “Violence” and “abuse” are both used to reflect that CPVA may not necessarily involve acts of physical violence. Gallego et al. (2019) summarize the various definitions, including some that refer to children and young people perpetrating physical violence or threats of physical violence, some which include one-off incidents, and a wide range of behaviors which have been described as psychological CPVA, including controversially, disobedience and shouting. In contrast, the umbrella term CPVA recognizes that this form of family violence has been associated with children and young people who themselves have experienced trauma and abuse.

## Aims and Research Questions

The aim of this scoping review is to present an overview of the diverse body of CPVA literature by identifying a broad range of studies, focusing on characteristics, risk and protective factors, and help-seeking behaviors. The scoping review questions are:

(1) What are the common characteristics of CPVA?
(2) What are the characteristics of children and young people in cases of CPVA?
(3) What are the characteristics of parents who experience CPVA?
(4) What are the risk and protective factors in families affected by CPVA?
(5) What are the barriers and facilitators to help-seeking in families where CPVA exists?

Although published scoping reviews on the topic of CPVA exist, these have been more narrowly focused on parenting, the family environment, and social support (Arias-Rivera et al., 2022); theoretical frameworks and explanatory factors (Arias-Rivera & García, 2020); measures of CPVA (Arias-Rivera et al., 2020); mapping CPVA within the larger field of childhood aggression (Rutter, 2023b); risk factors (Junco-Guerrero et al., 2024), and factors and developmental pathways of young people who engage in CPVA (Peck et al., 2021). Published systematic reviews have focused on concepts of CPVA (Holt, 2016a; Ibabe, 2020; Miles & Condry, 2015; Peck et al., 2023); integrating past CPVA research using a narrative approach (Simmons et al., 2018); CPVA interventions (Toole-Anstey et al., 2023a); and a meta-analytical review of the relationship between CPVA and child abuse (Gallego et al., 2019). There has been one rapid review focusing on characteristics of CPVA (Moulds & Day, 2017)

The focus of this scoping review differs and centers upon CPVA characteristics, risk and protective factors, and the barriers to and facilitators of help-seeking. This focus is broader than the existing published scoping and systematic reviews. Systematic reviews synthesize findings across all the reviewed studies. In contrast, this scoping review will survey the scope of the studies reviewed and will not attempt to synthesize results, evidence, or aggregate findings from different studies (Arksey & O’Malley, 2005). Instead, the extent, range, and nature of the research activity will be established, and gaps in the existing literature will be identified (Arksey & O’Malley, 2005). This approach will enhance the growing and emerging work on CPVA by offering directions for future research, policy, and practice.

## Method

This study employed a scoping review methodology (Arksey & O’Malley, 2005). To report the results of the scoping review, the authors adopted the Preferred Reporting Items for Systematic Reviews and Meta-Analyses (PRISMA) reporting guidelines (Page et al., 2021). The protocol was developed by Author 1 and was registered at the Open Science Framework on May 10, 2022 (https://doi.org/10.17605/OSF.IO/USJZE).

### Study Eligibility

The inclusion criteria were:

- Population: Families affected by CPVA;
- Concept: CPVA including cyber, economic, psychological, physical, sexual, and verbal abuse, coercive and controlling behaviors;
- Focus of study: Characteristics of children and young people, parents and carers, risk factors, protective factors, barriers to, and facilitators of help-seeking;
- Type of evidence: Primary original research and case studies published in peer-reviewed journals;
- English language studies as translation resources were not available for this project;

No studies were excluded on the basis of geographical location or year of the study. Scoping and systematic reviews were excluded on the basis that this would lead to double counting of many of the reports selected for the scoping review. We excluded gray literature as this is not peer-reviewed. Consideration was given to using the criteria in the Mixed Methods Appraisal Tool (Hong et al., 2018), however, the quality of the studies was not assessed or used as an exclusion criterion to ensure that the range of CPVA research is accurately mapped (Arksey & O’Malley, 2005).

### Study Selection and Data Extraction

Searches of the following databases took place in August 2023: CINAHL, Medline, PubMed, Scopus, and Web of Science. The search terms are presented in Table 1. All search terms were used for CINAHL, Medline, Scopus, and Web of Science. “CPVA” and “CAPVA” were omitted from the search on PubMed due to the large number of results relating to medical conditions with the same acronym. After the review and removal of 583 duplicate records manually, the remaining records were screened based on the title and abstract by both researchers. Any screening conflicts were resolved by the authors conferring. Initially, a broad-brush approach was taken so that any report about CPVA was carried forward to be assessed for eligibility. A large number of records were excluded at this stage (381 records) due to the use of acronyms denoting medical terms being captured in the database searches.

**Table 1.** Search Terms.

CPVA	Adolescent-to-parent violence
CAPVA	Adolescent-to-parent abuse
Child*-to-parent violence	Violence against parents*
Child*-to-parent abuse	

*Note.* CPVA = Child-to-parent violence and abuse; CAPVA = Child-and-adolescent-to-parent violence and abuse.

Handsearching of key journals was undertaken to identify articles which may have been missed in the database searches due to variations in coverage, indexing, and depth of information. Hand searches of the following journals were carried out in August 2023: *Journal of Family Violence*, *Journal of Interpersonal Violence*, and *Aggression and Violent Behavior*. The hand searches covered the period from January 2005 to August 2023 and resulted in seven additional papers being added to the scoping review.

In addition to hand searching, the list of citations from previous reviews from the main searches was also checked for new reports until a saturation point was reached when no new papers were identified (Arksey & O’Malley, 2005). A further 14 reports were identified using this method.

After the completion of the database searches, hand searching, and citation checking, the full texts of the remaining 286 reports were obtained for review. A full-text screening of these reports against the research questions and inclusion criteria determined the final set of 145 included reports. Reports were included if the sample data, or part of the sample data, included children and young people under the age of 18. There were some examples of reports of studies with university students, which were included as the study focused on their experiences of childhood. The reasons for exclusion at this stage were as follows: 50 records were excluded due to no English language record being available; 39 records were excluded due to not being relevant to the scoping questions; and 51 records were excluded on the basis of type of record (e.g., magazine articles, discussion pieces, and other scoping and systematic reviews).

An extraction table was developed in Microsoft Excel to assist with charting the data (Arksey & O’Malley, 2005). Key information about the reports was extracted, including author(s), year of publication, study location, method, sample characteristics, year of study, theory or model of CPVA, main findings, and variables and limitations. Further Excel sheets were compiled for more detailed charting of reports, including whether the study addressed characteristics of CPVA, risk, and protective factors, barriers to and facilitators of help-seeking. It was not always possible to extract all the information needed from every study as not all research reports detailed uniform data or material relevant to this scoping study.

**Figure 1.**
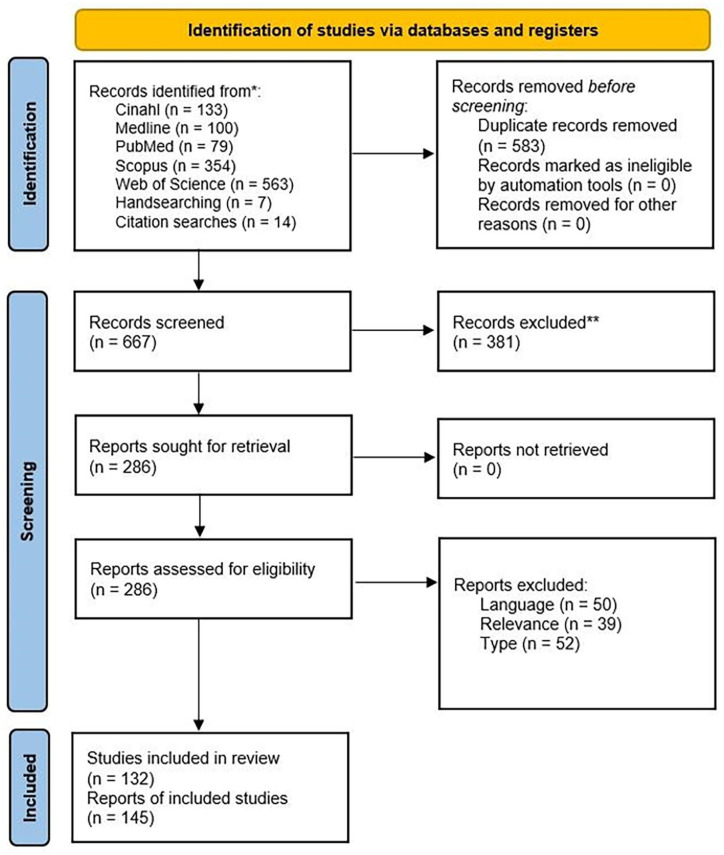
PRISMA flow diagram.

Figure 1 is the PRISMA flow diagram for this scoping review, illustrating the identification of studies via databases and registers. The difference between the number of studies and reports (±13) is due to some studies producing more than one published report.

## Results

This section of the paper begins with a description of included studies. Then, to reflect the scoping review questions, the subsequent section is split under the following headings: common characteristics of CPVA; characteristics of children and young people in cases of CPVA; characteristics of parents who experience CPVA; risk and protective factors for CPVA; barriers to and facilitators of help-seeking for CPVA. For clarity, the rest of this paper will refer to authors and studies rather than reports, as the charting of the field of CPVA is explored in more detail. Please refer to the Supplemental file accompanying this paper for a summary of all included reports.

### Description of Included Studies

**Figure 2.**
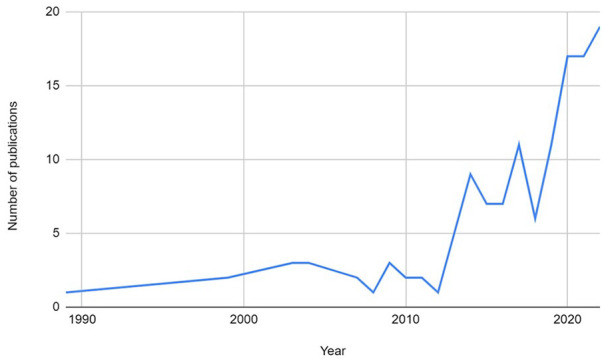
Number of child-to-parent violence and abuse reports by year.

The final set of records was published between 1989 and August 2023, with data collection dates ranging from 1972 to 2021 (where this was explicitly stated in the study). It was interesting to note that, overall, the field of CPVA is rapidly growing. Figure 2 illustrates the increase in published reports on CPVA, identifying in particular the rapid growth of the topic within the last decade, where publishing has roughly doubled. The figures are taken from the number of reports from the database searches, hand searches, and citation checking, and before the exclusion of reports based upon the criteria for this specific scoping review (the total number of reports about CPVA in any given year, regardless of focus or type of report).

**Figure 3.**
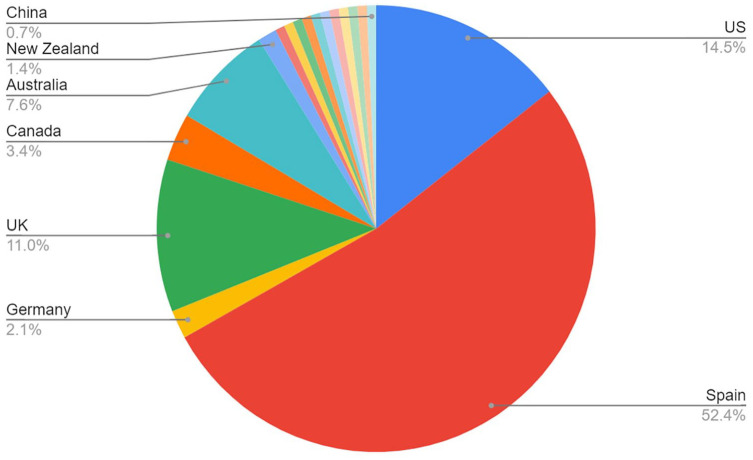
Location of reports.

Figure 3 is a pie chart visualizing the location of the reports based on the academic institution of the authors disclosed in the report. Where there are multiple authors from academic institutions in different countries, the first author’s academic institution has been counted. This clearly demonstrates that in the final set of 145 reports, the majority of included reports are from academic institutions located in Spain (52.4%). The next biggest number of reports are from the United States (14.5%) and the United Kingdom (11%). The overall percentage share of reports from research conducted in Spain is also an underestimation, as a further 50 reports were excluded from the final set due to there not being an English language translation available; the language of the majority of these reports was Spanish. The possible reasons for the dominance of Spanish studies on the topic of CPVA are further explored below.

**Figure 4.**
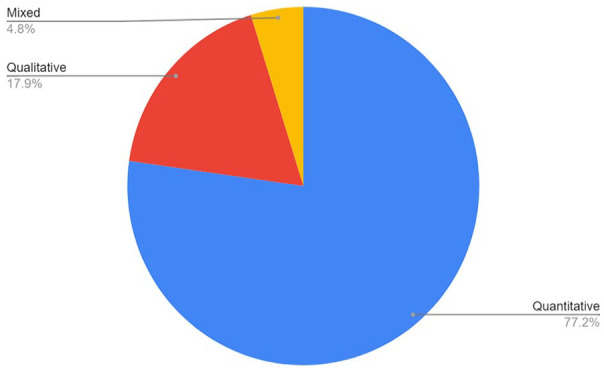
Research methods.

The split of the reports in terms of qualitative, quantitative, or mixed research methods demonstrates a strong preference for quantitative methods in the field of CPVA research. Figure 4 shows that 77.2% of reports applied quantitative methods, with a much smaller proportion of studies applying qualitative (17.9%) and mixed (4.8%) methods.

**Figure 5.**
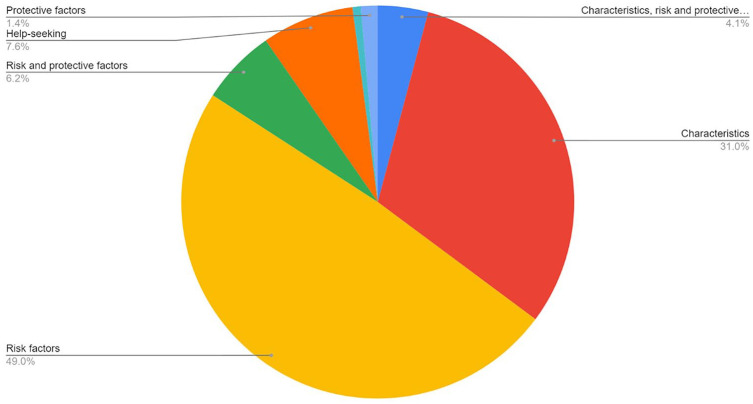
Focus of reports.

Within the included studies, the sample characteristics were either children and young people drawn from educational institutions, police, and judicial data, or clinical data. Some studies set no minimum age limit (*n* = 4). Only a handful of studies excluded children and young people by gender: studies looking at males only (*n* = 5) and studies looking at females only (*n* = 2). The vast majority of CPVA studies had mixed gender samples; however, this was often described as a binary category (male/female) or not specified whether this included nonbinary young people. Similarly, few studies excluded the parent or carer by gender: studies looking at mothers and grandmothers only were modest in number (*n* = 8), and there were no studies looking at fathers only. Finally, for the purposes of this scoping review, reports were also categorized according to the focus of the report under the following headings: characteristics, risk/protective factors, and help-seeking. The overwhelming majority of reports (49.7%) focused on identifying CPVA risk factors, followed by characteristics (of CPVA or the child/parent/family). Relatively few reports focusing on help-seeking (7.6%) were identified (see Figure 5).

### Common Characteristics of CPVA

Of the studies focusing on the characteristics of CPVA, the four most common types of CPVA identified were psychological CPVA (*n* = 16), physical CPVA (*n* = 15), financial CPVA (*n* = 7), and control over parents (*n* = 11). Several studies used a questionnaire (CPV-Q) to measure CPVA, which was developed in Spain (Calvete, Gamez-Guadix et al., 2013; Contreras et al., 2019, 2020). Other studies took a qualitative approach, identifying psychological, physical, and financial characteristics of CPVA through parent and carer experiences via analysis of interviews (Clarke et al., 2017; Eckstein, 2004) or a parenting website message board (Holt, 2011).

Other characteristics of CPVA identified in studies to a lesser extent were: CPVA involving weapons (*n* = 4) (Liettu et al., 2012; Moen & Shon, 2021; Walsh & Krienert, 2007, 2009); CPVA as an escalating pattern of violence and abuse (*n* = 1) (Eckstein, 2004); CPVA involving property damage (*n* = 1) (Murphy-Edwards & van Heugten, 2018); and cyber CPVA (*n* = 1) (Suárez-Relinque & del Moral-Arroyo, 2022). Of all the studies focusing on identifying characteristics of CPVA, most of the sampled population was from the community, with the exception of Moen and Shon (2021), Clarke et al. (2017), Liettu et al. (2012) and Walsh and Krienert (2007), where the child/young person in the study was also known to youth justice and/or the police.

### Characteristics of Children and Young People in Cases of CPVA

A total of 52 studies focused on the characteristics of children and young people in cases of CPVA. The largest count of studies were on the following characteristics: gender of the child or young person (*n* = 28); psychological, psychosocial, or personality characteristics (*n* = 19); and age (*n* = 18). Of the 18 studies focusing on the characteristic of age, over half (*n* = 9) used police and crime data, with some upper age limit cut-offs of 18 (Armstrong et al., 2021; Schut et al., 2020) or 21 (Walsh & Krienert, 2007). Other studies used high school cohorts in their sampling procedure, leading to lower cut-off ages of 12 (Calvete et al., 2022; Navas-Martínez & Cano-Lozano, 2023; Suárez-Relinque et al., 2023), 13 (Calvete et al., 2013, 2020) and 14 (Martin & Cortina, 2023).

Other characteristics of children and young people explored by authors in the included studies were ethnicity (*n* = 11), offending behavior (*n* = 8), substance misuse (*n* = 6), and mental health difficulties (*n* = 4). No studies included in the final set for this scoping review foregrounded disability, even though this was not an exclusion criterion of this review.

### Characteristics of Parents Who Experience CPVA

There were fewer studies focusing on the characteristics of parents who experience CPVA. The largest number of studies reported on the gender characteristics of the parent (*n* = 20). Of the studies concerning the gender of the parent as a variable, eight found that mothers were more likely to experience CPVA (Calvete et al., 2013; Contreras & Cano, 2014a; Lyons et al., 2015; Pagani et al., 2004; Rico et al., 2017; Schut et al., 2020; Ulman & Straus, 2003; Walsh & Kreinert, 2007). In two studies, gender was not a statistical variable; however, in the respective samples, it was found that mothers were more likely to be the victim of CPVA or parricide (Laurent & Derry, 1999; Moen & Shon, 2021). A further two studies found that mothers experienced more psychological CPVA and fathers more physical CPVA (Del Hoyo-Bilbao et al., 2018; Romero-Méndez et al., 2021). An additional two studies found that there were no (victim) gender differences in the prevalence; however, mothers experienced more episodes of CPVA over time (Calvete Gamez-Guadix et al., 2013; Contreras et al., 2020). A single study found no differences between the frequency of CPVA between mothers and fathers, although consideration was given to the increased presence of fathers in the home due to COVID-19 as the data collection timeframe included the first three waves of the pandemic (Calvete et al., 2022). The remaining studies were either qualitative (Cottrell & Monk, 2004; Messiah & Johnson, 2017), or measured how the gender of the parent affected other aspects of CPVA (Gebo, 2017; Margolin & Baucom, 2014).

There were six studies which described parenting or marital status as a characteristic identifying single-parent status, divorce or separation (Contreras & Cano, 2014a, 2014b; Gebo, 2007; Messiah & Johnson, 2017; Routt & Anderson, 2011). Other parent characteristics identified in two or fewer studies were socioeconomic class or family income (*n* = 2) (Routt & Anderson, 2011, Sheed et al., 2023a) and age (*n* = 1) (Calvete et al., 2022). Two studies found that CPVA occurs disproportionately in families of low socioeconomic backgrounds and in single-parent families (Gebo, 2007; Sheed et al., 2023a).

### Risk Factors for CPVA

A total of 85 studies explored risk and protective factors for CPVA. These studies identified a diverse set of 39 risk factors associated with CPVA. The most common risk factors identified in the final set of studies were previous domestic violence and/or abuse (DVA) within the family (*n* = 43); parenting practices or parenting style, for example, authoritative parenting, neglect or corporal punishment (*n* = 33); and previous physical child abuse (*n* = 32). Further risk factors for young people included family conflict/stress/problematic communication as a risk factor for CPVA (*n* = 22); alcohol or substance misuse by the child or young person (*n* = 19); the mental health or psychological factors experienced by the child or young person (*n* = 17); risk factors related to school behavior, peer groups and/or involvement in antisocial behavior (*n* = 17); and “delinquency” or offending behavior of the child or young person as a risk factor (*n* = 14).

Smaller numbers of papers identified child sex abuse (*n* = 6), gang association or street violence experienced by the child or young person (*n* = 5); emotional problems experienced by the child or young person (*n* = 5); the adverse childhood experiences or complex trauma of the child or young person (*n* = 4); impulsive behavior of the child or young person (*n* = 4); peer bullying (*n* = 4); childhood aggression (*n* = 3); a child or young person having an intellectual or physical disability (*n* = 2); and problematic social media use or cyberbullying (*n* = 2).

Other family risk factors included verbal child abuse (*n* = 10); divorce or being a single-parent or carer as a risk factor (*n* = 9); attachment problems (to parent or carer) or parental rejection as a risk factor (*n* = 6), smaller families (*n* = 3); alcohol or substance misuse by the parent (*n* = 3); mental health of the parent (*n* = 2); parent education level (*n* = 2); poverty (*n* = 2); academic failure or school refusal (*n* = 2); and, conversely, a higher family income as a risk factor (*n* = 2); and familial social isolation (*n* = 2).

Finally, the following risk factors were identified in one study each: mothers who had experienced child sex abuse, offending behavior of the parent or carer, third-party involvement in CPVA incidents, weapons, Covid-19, alexithymia (a neuropsychological condition resulting in the inability to recognize or describe one’s own emotions) and previous unsuccessful interventions (See Supplemental file for specific details of risk factors and studies).

### Protective Factors for CPVA

A number of studies (*n* = 13) focused on identifying protective factors for CPVA. Family cohesion and/or positive family relationships were the most common protective factor (*n* = 7); followed by open family communication (*n* = 2) (Jimenez et al., 2019; Suárez-Relinque et al., 2020); and school support or a positive classroom environment (Beckmann, 2020b; Ibabe et al., 2013). Single studies identified targeted intervention (Navas-Martínez & Cano-Lozano, 2022), positive peer relationships (Nam et al., 2022), and playing violent video games were associated with lower levels of CPVA (Ruiz-Fernandez et al., 2021).

### Barriers to and Facilitators of Help-Seeking for CPVA

In comparison to the overall number of studies in this scoping review, very few studies focused on barriers to and facilitators of help-seeking for CPVA. Of the 13 “help-seeking” studies, 12 were qualitative, and a single study used mixed methods, contrasting with the dominance of quantitative studies focusing on risk factors. The largest number of studies identified barriers relating to a lack of practitioner awareness or understanding (*n* = 8) and parent/carer feelings of guilt, shame, and hopelessness (*n* = 7). Other barriers identified were mother/parent blaming by practitioners (*n* = 3), lack of services (*n* = 2), fear and fear of family separation (*n* = 2), and a lack of multiagency frameworks or pathways for CPVA (*n* = 2). Single studies identified a lack of understanding from family members (*n* = 1) and living in a small community or rural location (*n* = 1).

In terms of identifying facilitators of help-seeking, three studies identified peer support as a facilitator (Clarke et al., 2017; Correll et al., 2017; Edenborough et al., 2008), and two studies identified crisis intervention services (Edenborough et al., 2008; Sporer & Radatz, 2017). Single studies identified engaging with activities outside of the home (Clarke et al., 2017), participating in a CPVA intervention (Correll et al., 2017), participating in counseling (Edenborough et al., 2008), a pre-existing relationship with a practitioner (Toole-Anstey et al., 2023b); removing the child/young person from the home (Sporer & Radatz, 2017); and early intervention (Edenborough et al., 2008).

## Discussion

There has been a rapid expansion in the field of CPVA research, with published reports doubling in the last decade (Figure 3—Number of CPVA reports by year); see Table 2 and 3 for a summary of our findings and the implications for policy, practice and research. However, a number of methodological and conceptual issues present challenges for scoping and systematic reviews, as well as CPVA research more broadly. For example, more than three-quarters of the reports in our review used quantitative methods only (77.2%), drawing sharply into focus the pressing need for more qualitative understanding of this topic. Moreover, CPVA literature has previously been described as complex, and along with the absence of an agreed definition, the existing body of research on CPVA displays a significant variation in terminology and operationalized concepts between prevalence estimates and research findings (Arias-Rivera & García, 2020; Simmons et al., 2018). Two frequently cited CPVA definitions used in empirical studies are: “any act of a child that is intended to cause physical, psychological or financial damage in order to gain control over a parent” (Cottrell, 2004) and “a pattern of behavior that uses verbal, financial, physical or emotional means to practice power and exert control over a parent” (Holt, 2013). It has been theorized that the difference in these definitions, when operationalized for quantitative research into prevalence, could explain the differences in prevalence estimates in the Global North (Holt, 2016a).

**Table 2.** Critical Findings.

• The field of CPVA is rapidly growing, doubling in the last decade. • There is no agreed operational definition of CPVA, leading to considerable variations in population sampling by age, prevalence rates, and research evidence. • Children and young people with disabilities, or who identify as transgender or nonbinary gender, or who are adopted or fostered are almost completely absent from the existing research. • Similarly, there is little research examining diversity in terms of ethnicity, culture, religion, language, nationality, or immigration status in terms of child, parent and/or family characteristics. • There is a plethora of studies describing risk factors but very limited research examining protective factors or help-seeking.

*Note.* CPVA = Child-to-parent violence and abuse.

**Table 3.** Implications for Policy, Practice, and Research.

• Policy and practice in relation to CPVA need to reflect its complexity and address the numerous risk factors identified within the literature. • This review found that DVA was a common risk factor for CPVA; therefore, policy and practice responses should recognize that CPVA can be a consequence of DVA rather than a form of DVA to avoid pathologizing children and young people. • More than three-quarters of the studies in this research employed quantitative methods, highlighting the need for more qualitative understanding of CPVA. This is in relation to the experiences, perceptions, and views of children, young people, parents, and carers and in relation to relevant practitioners from health, social care, education, and criminal justice to name a few. • Qualitative research would facilitate more contextual knowledge, which is needed in relation to risk and protective factors to inform policy and practice in prevention, early help, assessment, and case management. • Marginalized identities and communities can have both the same and different risks, vulnerabilities, and needs; therefore, more research is needed to understand CPVA in families in relation to ethnicity, culture, religion, language, nationality or immigration status, same-sex families and other non-normative family types. • In addition, more research is needed to understand diverse and marginalized young people who experience mental ill health, learning, and physical disabilities, or who identify as transgender or nonbinary. • To enable evidence-informed responses from policy and practice, research is urgently needed which examines the barriers and facilitators to help-seeking for children and their parents and carers. • The predominance of research from Spain draws attention to the acute need for more research in other high-, middle- and low-income countries across the globe.

*Note.* CPVA = Child-to-parent violence and abuse; DVA = domestic violence and/or abuse.

Arias-Rivera and García (2020) discuss a definition proposed by experts from the Spanish Society for the Study of Child-to-Parent Violence where CPVA is:

Repeated acts of physical, psychological (verbal or nonverbal) or economic violence by children against their parents or parental figures. The following behaviors are not considered child to parent violence: one off acts of aggression, those perpetrated during a diminished state of awareness that are not repeated once said awareness is recovered (alcohol intoxication, withdrawal syndromes, delirium or hallucination), those caused by (transitory or permanent) psychological disorders (autism or severe mental disability) and parricide with no prior history of aggression. (Arias-Rivera & García, 2020, p. 220)

Arias-Rivera and García (2020) note, however, that there is no agreement regarding the exclusion of instrumental and reactive violence and abuse and the controversial absence of power and/or control as a feature of CPVA, which most authors emphasize as a defining trait. In addition, this definition does not incorporate the ways in which children and young people use digital and communication technologies to perpetrate abuse and exert power and control (known as cyber or digital violence), yet there is a burgeoning body of evidence that describes technology as a core means of perpetrating interpersonal violence (see, e.g., Rogers et al., 2022). This neglect reflects the findings of this review as only one study explored cyber violence as a form of CPVA (see Suárez-Relinque & del Moral-Arroyo, 2022).

A consensus is yet to emerge in CPVA research on the inclusion of younger children, with some studies excluding younger children via an age cut-off, or sampling high school children as representative of the target population. The range of approaches to population sampling to investigate CPVA speaks to the diversity of opinion about whether CPVA is strictly an *adolescent* phenomenon or whether younger children should be captured in definitions with considerations of how capable a younger child is of practicing power and exerting control over a parent (Holt, 2013). There is an additional question as to whether CPVA is distinct from early childhood aggression. As noted by Rutter (2023b), conducting scoping and systematic reviews remains challenging due to different definitions of CPVA and naming conventions in different disciplines (e.g., “adolescent” or “child”; “aggression” or “abuse”). In addition, not all study authors included a lower age limit, even when they offered an upper age limit. Similarly, the cut-off at the upper age of CPVA has varied, depending on whether “child” has been operationally defined as a chronological age (linked to the age of majority in the relevant jurisdiction) or as the relationship between the parent and child (Simmons et al., 2018). For this scoping review, studies focusing exclusively on adult children were excluded; however, studies including a mixed sample of children under and over 18 were included.

Holt (2016a) discusses the similarities and differences between CPVA and DVA and the extent to which CPVA research, theory, policy, and practice have been based upon established ways of working with DVA. In terms of similarities, CPVA can fit into both the “family conflicts” and “gender-based violence” paradigms dominant in DVA and interpersonal violence research (Holt, 2016a). Both Holt (2016a) and Selwyn and Meakings (2016) point to how the distinct *differences* between DVA and CPVA present challenges for practitioners when faced with mothers experiencing gendered, multiple forms of abuse and where service responses are child-welfare or child-abuse focused. Another difference is that services and practitioners may be concerned with the potential for children and young people to be criminalized under DVA legislation, which is aimed at adult perpetrators (Bettinson & Quinlan, 2020). For instance, legislation that defines DVA in England and Wales states that this can involve people who are personally connected, through family or intimacy, from the age 16 years of age; that is, a child aged 16 may be labeled as a perpetrator of DVA via this legislation.

To further complicate the picture, there is previous research on the bidirectionality of CPVA and co-occurring with other forms of family violence (Ibabe et al., 2009, 2013), and a large number of studies within this review highlight DVA and other forms of child abuse as risk factors for CPVA. It is the latter which opens up discussion about whether it is useful and appropriate to consider CPVA to be a consequence or symptom of DVA rather than a sub-form of violence in its own right. However, some authors continue to categorize CPVA as a “type” of DVA (Junco-Guerrero et al., 2022).

Other conceptual issues related to the relationship between CPVA and parricide. A decision was made not to exclude studies on parricide on the grounds of relevance, and subsequently, three parricide studies, or “parricide-including” studies, were captured in the searches for this scoping review. Holt (2017) questions the dominant theoretical assumption that CPVA (as non-fatal violence) and parricide (as fatal violence) are two separate phenomena. A different study explored attempted and completed parricides (Moen & Shon, 2021), and another took a broad focus on police incidents relating to child-initiated family violence (Walsh & Krienert, 2009), further illustrating the point that there is an argument of a continuum between CPVA and parricide. These debates highlight a need for more conceptual and theoretical work to understand the relationship between CPVA and parricide, not least to assist practitioners working with high-risk cases.

In terms of diversity, a limited number of CPVA studies included children with intellectual and physical disabilities. This reflects Rutter’s (2023a) study, which found there to be a limited exploration of disability in the CPVA literature. In addition, there are differences in terms of national contexts and the ways in which disability is defined (physical, intellectual, learning disability, mental health terms, and so on). In two other scoping reviews, children with disabilities were excluded on the grounds of not meeting their definition of CPVA and of the studies reviewed, it was noted that around one third of studies did not specify a CPVA definition (Arias-Rivera et al., 2020, 2022). This draws attention to the lack of research exploring CPVA and children with disabilities, which might be due to the complexities of researching a mode of abuse concerned with issues of power and control with a population of children who have less power and control than other populations of children; that is, those without disabilities. There was a modest number of studies which examined ethnicity (*n* = 11) and a lack of focus on culture, religion, language, nationality, or immigration status in terms of child, parent and/or family characteristics.

Of the studies focusing on gender, some found differences in the type of CPVA exhibited according to gender identity. Some studies have found that boys are more likely to commit CPVA (Sheed et al., 2023a). Other studies have found that girls exercised more psychological or verbal abuse (Beckmann et al., 2021; Margolin & Baucom, 2014). However, some studies in Spain did not find significant gender differences in physical violence and similarly found that psychological violence from girls toward their mothers was more frequent (Calvete et al., 2013, 2014, 2022).

Furthermore, within the studies focusing on gender characteristics, there was also a presumption of a binary gender category of male/female. For example, studies referred to “both sexes” (Suárez-Relinque et al., 2022) or “both genders” (Martín & Cortina, 2023). There was no explicit exploration of gender-diverse, nonbinary, or transgender young people and the phenomenon of CPVA. Only one study was found that explicitly stated that a category of nonbinary children and young people was included, although the authors did not expand on this in their description of the sample nor in their reporting of the findings and conclusions (Calvete et al., 2022).

Another study stated that “there was no sex information for two young people” (referring to a binary male/female category), and these young people were subsequently excluded from the analysis (Sheed et al., 2023a). Of course, this data could be simply missing from the data set through human error or, when faced with binary categories in organizational data collection or questionnaires, left blank if this does not match with the young person’s gender identity. In either case, as a consequence, gender-diverse, nonbinary, and transgender children and young people are not visible in the CPVA literature.

Published research on CPVA from Spanish institutions represents a significant contribution to this field and constitutes 52.4% of the total number of reports in this review, outweighing studies from the next biggest contributors, the United States and the United Kingdom, combined (25.5%). This large body of work has been attributed to a significant media and social impact, linked to the concern about criminal reporting of CPVA leading to one of the highest rates of increase of all crime in Spain (Cuervo & Palanques, 2022). This does not mean that CPVA is more prevalent in Spain, but that the large body of research has resulted in increased public awareness and reporting to statutory bodies. Moreover, this review finding (the predominance of Spanish research on CPVA) skews the scholarship in the field and highlights the need for research in other countries, particularly in low to middle-income countries, which are barely represented in terms of the geographical location of existing research.

Finally, across the sample of 145 reports, while a significant number of studies reported on characteristics of child/young persons and parents and examined risk factors, very few sought to describe protective factors or help-seeking.

### Limitations

There are several limitations to this scoping review. Firstly, although the intention was to cast a wide net to catch as many CPVA studies as possible, due to the wide range of alternative terms for CPVA, reports and studies will have been missed. For example, there are some less frequently used terms, such as “adolescent family violence,” “adolescent violence in the home” and “adolescent to mother violence” which were not included in the search terms. Overlapping terms such as “parent abuse” were not included as search terms for this scoping review due to the body of social work literature using this term to refer to abuse perpetrated by parents and carers. There was also some overlap in studies between what was defined as a characteristic of a child or young person engaging in CPVA and what constitutes a risk or protective factor. For example, a psychological profile of childhood aggression was described as both, as were mental health difficulties and disabilities. Use of weapons was both described as a characteristic of CPVA, and as a risk factor. For the purposes of this review, the studies’ authors’ usages were counted. Some characteristics and risk and protective factors were grouped together using the author’s judgment of whether the choice of language or differences in description within the study were minor.

Reviews predominantly drawing upon database searches will reflect the acknowledged United States, or “Western,” bias inherent in those databases (Arksey & O’Malley, 2005). Furthermore, the exclusion of non-English language studies was also due to the author’s limitations and will exacerbate existing biases. It is of note that even with the exclusion of non-English language studies, over half of the remaining studies originated in Spain (52.4%).

This scoping review also does not address how to synthesize findings from different kinds of study design or make quality judgments of the “weight” the different studies should carry. In exploring the breadth of the field of CPVA, depth has been sacrificed in this review. Future systematic reviews can address these shortcomings, contribute to the ongoing debate surrounding the conceptualization of CPVA as a phenomenon, and work toward an accepted definition and naming convention.

Finally, a consultation exercise with stakeholders was not conducted as part of this scoping review, as is recommended (Arksey & O’Malley, 2005). This was due to time and funding constraints; however, it could be realized in future CPVA scoping and systematic reviews.

## Conclusion

This review has established that the field of CPVA research is rapidly growing, and a consensus is yet to emerge on the conceptualization of CPVA and: the definition of CPVA as a social phenomenon; CPVA’s relationship to DVA; whether CPVA is exclusively an *adolescent* phenomenon; and whether CPVA includes children and young people with intellectual and/ or mental disabilities. Some children and young people, such as those with disabilities, who are gender non-conforming and who are adopted or fostered, are almost completely absent from the existing research. The existing quantitative research mostly relies upon police and criminal justice data or community samples, drawing almost exclusively on questionnaires completed with children and young people in high schools and their families. Future research could explore other organizational data or different measurement instruments to see to what extent the lack of consensus on CPVA prevalence and gender differences could be explained by biases within the sampling and existing measures of CPVA. The very limited research focusing on protective factors and help-seeking has significant implications for policy and strategic leadership of health services, the police and youth justice, social services, organizations in the community and voluntary sector, and the professionals and practitioners working within these organizations.

## Supplemental Material

sj-docx-1-tva-10.1177_15248380241246033 – Supplemental material for Child-to-Parent Violence and Abuse: A Scoping Review Supplemental material, sj-docx-1-tva-10.1177_15248380241246033 for Child-to-Parent Violence and Abuse: A Scoping Review by Michaela M. Rogers and Charlotte Ashworth in Trauma, Violence, & Abuse
