# Supplementary material for: Child-to-Parent Violence and Abuse: A Scoping Review
Source: Trauma Violence Abuse. 2024 Apr 29;25(4):3285–98. doi: 10.1177/15248380241246033 (PMC11370202; doi:10.1177/15248380241246033)
Supplement: sj-docx-1-tva-10.1177_15248380241246033 – Supplemental material for Child-to-Parent Violence and Abuse: A Scoping Review [file sj-docx-1-tva-10.1177_15248380241246033.docx]

**CPVA Summary of studies**

| **Study** | **Location** | **Research method** | **Sample** | **Relevance to research questions** | **Characteristics/ Risk & Protective factors/ Help-seeking barriers and facilitators identified** |
| --- | --- | --- | --- | --- | --- |
| Agnew and Huguley (1989) | US | Quantitative | n=1,395 (young people) | Characteristics, risk and protective factors | Offending behavior, ethnicity, perceived parenting style, attachment, peer influence |
| Armstrong, Cain, Wylie, Muftić & Bouffard (2018) | US | Quantitative | n=293 (young people; weighted sample n=4,196) | Characteristics, risk and protective factors | Age (child), ethnicity (child), gender (child), substance misuse, mental health, single parent, previous abuse, poor school behaviour |
| Armstrong, Muftić & Bouffard (2021) | US | Quantitative | n=1,113 (calls to police in a Midwestern state) | Characteristics | Age (child), ethnicity (child), gender (child), offending behaviour |
| Bartle-Haring, Slesnick & Carmona (2015) | US | Quantitative | n=161 (young people with alcohol/substance misuse issues) | Characteristics, risk and protective factors | Ethnicity (child), gender (child), alcohol/substance misuse (child) |
| Bautista-Aranda, Contreras & Cano-Lozano (2023) | Spain | Quantitative | n=1,868 (young people) | Risk factors | Previous domestic violence/abuse, previous physical abuse |
| Beckmann (2020a) | Germany | Quantitative | n=2,490 (young people) | Risk and protective factors | Gender, previous domestic violence/abuse, previous physical abuse, positive family relationships |
| Beckmann (2020b) | Germany | Quantitative | n=10,638 (young people) | Risk and protective factors | Previous domestic violence/abuse, school support |
| Beckmann, Bergmann, Fischer & Mößle (2021) | Germany | Quantitative | n=6,444 (young people) | Risk factors | Gender (child), previous physical abuse, previous verbal abuse, alcohol/substance misuse (child), mental health (child) |
| Bettinson & Quinlan (2020) | UK | Qualitative | n=9 (practitioners) | Characteristics | Psychological CPVA, control CPVA |
| Brezina (1999) | US | Quantitative | n=2,213 (males) | Risk factors | Physical abuse, family strain |
| Calvete, Orue, Bertino, Gonzalez, Montes, Padilla & Pereira (2014) | Spain | Qualitative | n=7 (mothers); n=4 (fathers); n=5 (young people); n=5 (professionals) | Risk factors | Previous domestic violence/abuse, substance misuse (child), mental health (child), parenting style, marital conflict |
| Calvete, Gámez-Guadix & Garcia-Salvador (2015) | Spain | Quantitative | n=1,272 (young people) | Characteristics | Psychosocial profile |
| Calvete, Orue & Gámez-Guadix (2013) | Spain | Quantitative | n=1,072 (young people) | Risk factors | Substance misuse (child), mental health (child), parenting style |
| Calvete, Orue, Fernández-González, Chang & Little (2020) | Spain | Quantitative | n=1,415 (young people) | Risk factors | Previous domestic violence/abuse, substance misuse (child) |
| Calvete, Orue & Gámez-Guadix (2015) | Spain | Quantitative | n=981 (young people) | Risk factors | Substance misuse (child) |
| Calvete, Gámez-Guadix, Orue, Gonzalez-Diez, Lopez de Arroyabe, Sampedro, Pereira, Zubizarreta & Borrajo (2013) | Spain | Quantitative | n=2,719 (young people) | Characteristics | Age (child), gender (child), gender (parent/carer), psychological CPVA, physical CPVA, financial CPVA, controlling CPVA |
| Calvete, Orue, Gámez-Guadix & Bushman (2015) | Spain | Quantitative | n=591 (young people) | Risk factors | Previous domestic violence/abuse, parenting style |
| Calvete (2023) | Spain | Quantitative | n=1,647 (young people) | Risk factors | Previous family violence, parenting style |
| Calvete, Jimenez-Granado & Orue (2022) | Spain | Quantitative | n=1,244 (young people) | Characteristics | Age (child), gender (child), ethnicity (child), age (parent/carer), gender (parent/carer) |
| Calvete, Orue, Gámez-Guadix, Hoyo-Bilbao, de Arroyabe and López (2015) | Spain | Qualitative | n=15 (young people known to youth offending) | Risk factors | Previous domestic abuse/violence, previous physical abuse (child), parenting style |
| Cano-Lozano, Rodríguez-Díaz, León & Contreras (2020) | Spain | Quantitative | n=1,599 (young people) | Risk factors | Substance misuse (child), parenting style, peer group influence |
| Cano-Lozano, León & Contreras (2021a) | Spain | Quantitative | n=1,543 (university students) | Characteristics | Age (child), gender (child), gender (parent/carer), psychological CPVA, physical CPVA, financial CPVA, controlling CPVA |
| Cano-Lozano, León & Contreras (2021b) | Spain | Quantitative | n=1,543 (university students) | Risk factors | Parenting style |
| Cano-Lozano, Navas-Martínez, Contreras (2021) | Spain | Quantitative | n=2,245 (university students) | Risk factors | Previous domestic violence/abuse, previous physical abuse, covid-19, psychosocial stressors |
| Cano-Lozano, Contreras, Navas-Martínez, León & Rodríguez-Díaz (2023) | Spain | Quantitative | n=208 (young offenders) | Risk factors | Previous domestic violence/abuse, previous physical abuse, previous verbal abuse (child), previous sexual abuse (child), parenting style, street violence, offending behaviour |
| Clarke, Holt, Norris & Nel (2017) | UK | Qualitative | n=6 (parents) | Characteristics | Offending behaviour (child), physical CPVA, psychological CPVA, financial CPVA, controlling CPVA |
| Condry & Miles (2014) | UK | Quantitative | n=1,892 (cases reported to the police) | Characteristics | Age (child), gender (child), ethnicity (child), offending behaviour (child) |
| Contreras, León & Cano-Lozano (2020a) | Spain | Quantitative | n=1,012 (parents/carers) | Characteristics | Gender (parent), physical CPVA, psychological CPVA, financial CPVA, controlling CPVA |
| Contreras & Cano (2015) | Spain | Quantitative | n=90 young people | Characteristics | Psychological profile, offending behaviour |
| Contreras & Cano (2014a) | Spain | Quantitative | n=90 (young people) | Characteristics, risk and protective factors | Gender (parent), parenting style, offending behaviour, single parent homes, small family size, higher family income |
| Contreras & Cano (2014b) | Spain | Quantitative | n=654 (young offenders) | Characteristics, risk and protective factors | Offending behaviour, single parent, previous domestic violence/abuse, previous physical abuse, parenting style, family conflict, small family, higher family income |
| Contreras & Cano (2016a) | Spain | Quantitative | n=90 (young people) | Risk factors | Previous family violence, physical abuse |
| Contreras & Cano (2016b) | Spain | Quantitative | n=60 (young people charged with an offence) | Risk factors | Social attitudes |
| Contreras, Bustos-Navarrete & Cano-Lozano (2019) | Spain | Quantitative | n=1,386 (young people) | Characteristics | Psychological CPVA, physical CPVA, financial CPVA, control CPVA |
| Contreras, León & Cano-Lozano (2020b) | Spain | Quantitative | n=1,624 (young people) | Characteristics | CPVA mechanisms |
| Correll, Walker & Edwards (2017) | US | Qualitative | n=15 (parents) | Help-seeking | Peer support, participation in an intervention |
| Cortina, Iribarren & Martín (2022) | Spain | Quantitative | n=127 (teachers) | Characteristics | Age (child), poor parenting, inadequate environment, emotional reaction, evil/madness (psychological trait) |
| Cortina & Martín (2020) | Spain | Quantitative | n=225 (high school students) | Characteristics | Psychological CPVA, physical CPVA, financial CPVA, control CPVA, substance misuse |
| Cortina & Martín (2021) | Spain | Quantitative | n=763 (young people and adults) | Risk factors | Poor parenting, inadequate environment, emotional reaction, evil/madness (psychological trait) |
| Cottrell & Monk (2004) | Canada | Qualitative | n=177 (parents, young people, professionals) | Characteristics, risk and protective factors, help-seeking | Gender (child), gender (parent), peer influence, parenting style, delinquency, school failure, substance misuse, mental health (child), mental health (parent), family conflict, previous domestic violence/abuse, previous physical abuse, poverty, isolation, parent shame |
| Cuervo (2023) | Spain | Quantitative | n=342 (young people) | Risk factors | Parenting style, negative relationship with parents |
| Cuervo and Palanques (2022) | Spain | Quantitative | n=341 (young people with a judicial record) | Characteristics, risk and protective factors | Gender (child), psychological factors (child), substance misuse, poverty, parenting style |
| Del Hoyo-Bilbao, Orue, Gámez-Guadix & Calvete (2020) | Spain | Quantitative | n=298 (young people) | Risk factors | Peer influence, parenting style, corporal punishment, impulsiveness, substance misuse |
| Del Hoyo-Bilbao, Gámez-Guadix & Calvete (2018) | Spain | Quantitative | n=96 (young people)] | Risk factors | Parenting style (corporal punishment) |
| Del Hoyo-Bilbao, Orue & Calvete (2022) | Spain | Quantitative | n=765 (young people) | Characteristics | Psychological profile |
| Del Hoyo-Bilbao, Gámez-Guadix, Orue, Calvete, Gámez-Guadix (2018) | Spain | Quantitative | n=169 (young people) | Characteristics | Gender (parent), psychological CPVA, physical CPVA |
| Del Moral, Suárez-Relinque, Callejas & Musitu (2019) | Spain | Quantitative | n=2,101 (young people) | Risk factors | Attitudes towards school |
| Eckstein (2004) | US | Qualitative | n=20 (parents) | Characteristics | Verbal CPVA, physical CPVA, psychological CPVA, escalating pattern CPVA |
| Edenborough, Jackson, Mannix & Wilkes (2008) | Australia | Qualitative | n=185 (mothers) | Help-seeking | Lack of awareness of CPVA (practitioners), support groups, peer mentoring, counselling, early intervention, crisis intervention |
| Murphy-Edwards, & van Heugten (2018) | New Zealand | Qualitative | n=14 (parents) | Characteristics | Property damage |
| Fandiño, Basanta, Sanmarco, Arce and Fariña (2021) | Spain | Quantitative | n=75 (young offenders) | Characteristics | Psychological factors, personality factors |
| Gabriel, Tizro, James, Cronin-Davis, Beetham, Corbally, Lopez-Moreno & Hill (2018) | UK | Qualitative | n=18 (young people, parents and practitioners) | Risk factors | Previous domestic abuse/violence, previous physical abuse, previous verbal abuse, alcohol/substance misuse, parenting style, social media |
| Galvani (2017) | UK | Qualitative | n=12 (practitioners) | Risk factors | Previous domestic violence/abuse, alcohol/substance misuse |
| Gebo (2007) | US | Quantitative | n=132 (young offenders) | Characteristics | Gender (child), gender (parent), offending behaviour, single parent |
| Harries, Curtis, Valpied, Baldwin, Hyder & Miller (2022) | Australia | Quantitative | n=1,132 (adults) | Risk factors | Parenting style (corporal punishment), physical abuse |
| Harries, Curtis, Skvarc, Walker & Mayshak (2022) | Australia | Quantitative | n=121 (parents/carers) | Characteristics | Physical CPVA, verbal CPVA, coercion CPVA |
| Hernández, Martín, Hess-Medler & García-García (2020) | Spain | Quantitative | n=148 (male young offenders) | Risk factors | Previous domestic violence/abuse, previous physical abuse, substance misuse, street violence, offending behaviour, school behaviour |
| Herrera and McCloskey (2003) | US | Qualitative | n=141 (mother and daughter pairs) | Risk factors | Previous domestic violence/abuse, previous physical abuse (child), previous sexual abuse (child) |
| Holt (2017) | UK | Quantitative | n=693 (recorded cases of parricide) | Characteristics | Age (child), gender (child), ethnicity (child) |
| Holt (2011) | UK | Qualitative | n=33 (online forum posts) | Characteristics | Emotional terrain of CPVA, psychologisation of the young person, parental responses |
| Holt & Birchall (2022) | UK | Qualitative | n=36 (grandparents and practitioners) | Help-seeking | Lack of understanding of CPVA (practitioners), fear of separation (from child) |
| Holt & Lewis (2021) | UK | Mixed | n=226 (practitioners) | Risk factors | Previous domestic violence/abuse, mental health (child), mental health (parent), sexual abuse (child), alcohol/substance misuse (parent), parenting style, ACES/complex trauma, complex needs, learning disability, previous verbal abuse |
| Ibabe, Jaureguizar & Bentler (2013a) | Spain | Quantitative | n=687 (high school students) | Protective factors | Positive family environment, positive classrooom environment, psychological factors |
| Ibabe (2019) | Spain | Quantitative | n=586 (high school students and their parents) | Risk factors | Parenting style, family conflict |
| Ibabe (2016) | Spain | Quantitative | n=584 (high school students) | Risk factors | Parenting style, parent education level, academic failure |
| Ibabe, Arnoso & Elgorriaga (2014a) | Spain | Quantitative | n=231 (young people and young offenders) | Characteristics | Psychological factors, offending behaviour |
| Ibabe, Arnoso & Elgorriaga (2014b) | Spain | Quantitative | n=231 (young people and young offenders) | Characteristics | Mental health, offending behaviour |
| Ibabe & Bentler (2019) | Spain | Quantitative | n=585 (high school students) | Risk factors | Parenting style |
| Ibabe & Jaureguizar (2010) | Spain | Quantitative | n=103 (young people) | Risk factors | Single parent family, smaller family size |
| Ibabe, Jaurequizar & Díaz (2009) | Spain | Quantitative | n=103 (young offenders) | Risk factors | Family violence |
| Ibabe (2014) | Spain | Quantitative | n=485 (young people) | Risk factors | Family violence |
| Ibabe, Jaureguizar & Bentler (2013b) | Spain | Quantitative | n=485 (young people) | Risk factors | Previous domestic violence/abuse, previous physical abuse, inappropriate parenting, substance misuse (parent), social maladjustment |
| Ilabaca Baeza & Gaete Fiscella (2021) | Chile | Quantitative | n=1,861 (young people) | Characteristics | Gender (parent), single parent |
| Jimenez, Estevez, Velilla, Martin-Albo & Martinez (2019) | Spain | Quantitative | n=2,399 (high school students) | Risk and protective factors | Family stress, problematic family communication, open communication |
| Izaguirre & Calvete (2017) | Spain | Quantitative | n=845 (young people) | Risk factors | Previous domestic violence/abuse, history of physical abuse |
| Jaureguizar, Ibabe & Straus (2013) | Spain | Quantitative | n=687 (young people) | Risk and protective factors | Positive family relationships, offending behaviour, antisocial behaviour |
| Jimenez-Garcia, Perez, Contreras & Cano-Lozano (2020) | Spain and Chile | Quantitative | n=905 (young people) | Characteristics | Gender (parent), psychological CPVA, physical CPVA, financial CPVA, control CPVA |
| Jimenez-Granado, Hoyo-Bilbao & Fernandez-González (2023) | Spain | Quantitative | n=671 (young people) | Characteristics | Psychological factors, psychological CPVA and physical CPVA |
| Junco-Guerrero, Ruiz-Fernández & Cantón-Cortés (2021) | Spain | Quantitative | n=904 (high school students) | Risk factors | Previous domestic abuse/violence |
| Kennedy, Edmonds, Dann & Burnett (2010) | US | Quantitative | n=223 (young offenders) | Risk factors | Mental health (child), gang membership/affiliation, mental health problems (child), previous suicide attempt, single parent/divorce |
| Laurent & Derry (1999) | France | Mixed | n=645 (children hospitalised in a psychiatry department) | Characteristics | Psychological factors, mental health (child), age (child), gender (child), gender (parent) |
| Liettu, Säävälä, Hakko, Räsänen & Joukamaa (2012) | Finland | Quantitative | n=192 (male young offenders) | Characteristics | Weapons |
| Loinaz & Ma-de-Sousa (2020) | Spain | Quantitative | n=91 (young people) | Risk factors | Previous domestic abuse/violence, previous physical abuse, mental health (child), mental health (parent), family conflict, offending behaviour (parent), offending behaviour (child) |
| Loinaz, Barboni, & Ma-de-Sousa (2020) | Spain / Uruguay / Netherlands | Quantitative | n=91 (young people) | Risk factors | Previous domestic abuse/violence, previous physical abuse, substance misuse (child), family conflict, low self esteem |
| Loinaz, Irureta & San Juan (2023) | Spain | Quantitative | n=206 (young people and adults) | Risk factors | Previous domestic violence/abuse, peer bullying, antisocial behaviour, school behaviour, family conflict, previous unsuccessful interventions |
| López-Martínez, Montero-Montero, Moreno-Ruiz & Martínez-Ferrer (2019) | Spain | Quantitative | n=1,200 (school children) | Risk and protective factors | Family conflict, family cohesion |
| López-Martínez, Montero-Montero, Moreno-Ruiz & Martínez-Ferrer (2021) | Spain | Quantitative | n=1,304 (high school students) | Risk factors | Peer bullying, cyberbullying |
| Lyons, Bell, Fréchette & Romano (2015) | Canada | Quantitative | n=365 (university students) | Risk factors | Previous domestic violence/abuse, previous physical abuse, previous verbal abuse (child), parenting style |
| Maclean (2016) | UK | Qualitative | n=1 (case study) | Risk factors | Neglect, anti social behaviour, family violence, offending behaviour |
| Maranon & Ibabe (2022) | Spain | Quantitative | n=389 (young people and parents) | Risk factors | Psychological factors, parenting style |
| Margolin & Baucom (2014) | US | Quantitative | n=93 (young people) | Risk factors | Previous domestic violence/abuse, previous physical abuse |
| Martín and Cortina (2023) | Spain | Quantitative | n=341 (high school students) | Characteristics | Age (child), gender (child), psychosocial factors (child) |
| Martín, De la Fuente, Hernández, Zaldívar, Ortega-Campos & García-García (2022) | Spain | Quantitative | n=89 (male young offenders) | Risk factors | Psychosocial factors |
| Martínez-Ferrer, Romero-Abrio, León-Moreno, Villarreal-González & Musitu-Ferrer (2020) | Spain | Quantitative | n=8,115 (high school students) | Risk factors | Psychological factors, mental health (child), low self-belief (child) |
| Martínez-Ferrer, Romero-Abrio, Moreno-Ruiz & Musitu (2018) | Spain | Quantitative | n=2,399 (high school students) | Risk factors | Cyberbullying, alexithymia |
| Messiah & Johnson (2017) | Trinidad | Qualitative | n=13 (family members) | Characteristics | Gender (parent), single parent, psychological CPVA, physical CPVA, control CPVA |
| Miles & Condry (2016) | UK | Mixed | n=100 (case files); n=20 (police officers) | Characteristics | Psychological CPVA, physical CPVA, financial CPVA, control CPVA |
| Moen & Shon (2021) | South Africa | Mixed | n=58 (offenders) | Characteristics | Ethnicity (child), ethnicity (parent), gender (child), gender (parent), weapon |
| Moulds, Mayshak, Mildred & Miller (2019) | Australia | Quantitative | Overall count not given | Characteristics | Age (child), gender (child), ethnicity (child) |
| Nam, Kim, Bright & Jang (2022) | South Korea | Quantitative | n=709 (young people) | Risk and protective factors | Previous domestic violence/abuse, peer relationships |
| Navas-Martínez & Cano-Lozano (2023a) | Spain | Quantitative | n=3,142 (high school students) | Risk factors | Adverse childhood experiences |
| Navas-Martínez & Cano-Lozano (2023b) | Spain | Quantitative | n=1,559 (high school students) | Risk factors | Previous victimisation, parenting permissiveness, emotional intelligence |
| Navas-Martínez & Cano-Lozano (2022c) | Spain | Quantitative | n=3,142 (high school students) | Risk factors | Previous domestic violence/abuse, previous physical abuse, previous verbal abuse, bullying |
| Navas-Martínez & Cano-Lozano (2022a) | Spain | Quantitative | n=1,559 (young people) | Risk and protective factors | Previous domestic violence/abuse, previous physical abuse, targeted intervention |
| Navas-Martínez & Cano-Lozano (2022b) | Spain | Quantitative | n=1,559 (young people) | Risk factors | Previous domestic abuse/violence, previous physical abuse, bullying, attachment issues, less emotional resilience |
| Nowakowski-Sims & Rowe (2017) | US | Quantitative | n=80 (young offenders) | Risk factors | Previous domestic violence/abuse, previous physical abuse, previous verbal abuse, previous sexual abuse |
| Nowakowski-Sims (2019) | US | Quantitative | n=83 (young people) | Risk factors | Previous domestic violence/abuse, previous physical abuse, previous verbal abuse, previous sexual abuse |
| Nowakowski & Mattern (2014) | US | Quantitative | n=209 (young offenders) | Risk factors | Previous arrest, problems in school |
| Orue, Calvete & Fernández-González (2021) | Spain | Quantitative | n=903 (high school students) | Characteristics | Maladaptive behaviour, social information processing issues |
| Pagani, Tremblay, Nagin, Zoccolillo, Vitaro & McDuff (2004) | Canada | Quantitative | n=1,175 (young people) | Risk factors | Corporal punishment, verbal punishment, childhood aggression |
| Pagani, Tremblay, Nagin, Zoccolillo, Vitaro & McDuff (2009) | Canada | Quantitative | n=774 (young people) | Risk factors | Parenting practices, parent education level, family structure, childhood aggression, substance misuse (child) |
| Pagani, Larocque, Vitaro & Tremblay (2003) | Canada | Quantitative | n=778 (young people) | Risk and protective factors | Family conflict, divorce, positive family relationship |
| Palanques, Cuervo & Villanueva (2022) | Spain | Quantitative | n=341 (young offenders) | Risk factors | Offending behaviour |
| Papamichail & Bates (2022) | UK | Qualitative | n=8 (young people) | Risk factors | Adverse childhood experiences, difficult relationships with parents, perceived parental rejection, emotional dysregulation |
| Peck, Hutchinson & Provost (2022) | Australia | Quantitative | n=775 (young people) | Risk factors | Previous domestic violence/abuse, previous sexual abuse (child), family conflict, anti social behaviour, adverse childhood experiences |
| Peck, Provost, East & Hutchinson (2023) | Australia | Quantitative | n=775 (young people) | Risk factors | Previous domestic abuse/violence, alcohol/substance misuse (child), alcohol/substance misuse (parent), neglect, mental health problems (child) |
| Pritchett & McGarry (2022) | UK | Mixed | n=20 (young people) | Help-seeking | Lack of awareness of CPVA (practitioners), lack of multi agency framework |
| Rico, Rosado & Cantón-Cortés (2017) | Spain | Quantitative | n=934 (high school students) | Risk factors | Impulsiveness |
| Romero-Méndez, Rojas-Solís, Amador (2021) | Mexico | Quantitative | n=450 (high school students) | Characteristics | Gender (parent), psychological CPVA, physical CPVA |
| Rosado, Rico & Cantón-Cortés (2017) | Spain | Quantitative | n=855 (high school students) | Risk factors | Psychopathy |
| Routt & Anderson (2011) | Australia | Quantitative | n=1339 (incidents) | Characteristics | Age (child), gender (child), ethnicity (child), marital status (parent), family income |
| Ruiz-Fernandez, Junco-Guerrero & Cantón-Cortés (2021) | Spain | Quantitative | n=916 (high school students) | Protective factors | Violent video games |
| Rutter (2021) | UK | Qualitative | n=6 (mothers) | Risk factors | Previous domestic violence/abuse, family conflict, emotional difficulties (child) |
| Rutter (2023a) | UK | Qualitative | n=6 (mothers) | Help-seeking | Mother-blaming, lack of services, lack of multi agency pathway |
| Sasaki, Usami, Sasaki, S., Sunakawa, Toguchi, Tanese, Saito, Shinohara, Kurokouchi, Sugimoto, Hakoshima, Inazaki, Yoshimura, Mizumoto & Okada (2021) | Japan | Quantitative | n=822 (clinical patients) | Risk factors | Previous domestic abuse/violence, previous physical abuse, previous verbal abuse, disability, mental health (child), anti social behaviour, school refusal, impulsivity |
| Schut, Sorenson & Gelles (2020) | US | Quantitative | n=2,361 (incidents) | Characteristics | Age (child), gender (child), ethnicity (child), gender (parent) |
| Seijo, Vázquez, Gallego, Gancedo & Novo (2020) | Spain | Quantitative | n=210 (high school students) | Characteristics | Gender (child), psychological CPVA, psychological adjustment |
| Selwyn & Meakings (2016) | UK | Mixed | n=390 surveys / 90 families interviewed | Help-seeking | Lack of awareness/understanding, parenting-blaming, feelings of shame |
| Sheed, McEwan, Simmons, Spivak & Papalia (2023) | Australia | Quantitative | n=5,014 (young people reported to the police) | Characteristics | Age (child), gender (child), ethnicity (child), mental health (child), socioeconomic class |
| Sheed, Maharaj, Simmons, Papalia & McEwan (2023) | Australia | Quantitative | n=82 (young people reported to police) | Risk factors | Substance misuse (child), mental health (child), neurodevelopmental issues (child), family conflict, weapons, school behaviour, previous abuse (type not specified), third party involvement/presence |
| Soto, Doménech & Mateo (2022) | Spain | Qualitative | n=10 (families) | Risk factors | Problematic communication |
| Sporer (2019) | US | Qualitative | n=4 (families) | Help-seeking | Removing the child from the home, crisis intervention, practitioner awareness/understanding, feelings of guilt |
| Sporer & Radatz (2017) | US | Qualitative | n=26 (mothers) | Help-seeking | Lack of practitioner understanding, lack of family understanding, lack of services, feelings of hopelessness |
| Suárez-Relinque & del Moral-Arroyo (2023) | Spain | Qualitative | n=12 (experts) | Characteristics | Cyber CPVA |
| Suárez-Relinque, del Moral Arroyo, Leon-Moreno & Callejas Jeronimo (2019) | Spain | Quantitative | n=2,112 (high school students) | Risk factors | Parenting style |
| Suárez-Relinque, del Moral Arroyo, Leon-Moreno & Callejas Jeronimo (2020) | Spain | Quantitative | n=3,713 (high school students) | Risk and protective factors | Problematic use of social networking sites, problematic communication (with parents), open communication (with parents) |
| Suárez-Relinque, del Moral Arroyo, Leon-Moreno & Callejas Jeronimo (2023) | Spain | Quantitative | n=1,928 (high school students) | Risk factors | Emotional loneliness, mental health (child), alexithymia |
| Toole-Anstey, Townsend & Keevers (2023) | Australia | Qualitative | n=20 (mothers and practitioners) | Help-seeking | Feelings of fear, shame and judgment, pre-existing relationship with a practitioner, rural location, small community |
| Toole-Anstey, Townsend & Keevers (2022) | Australia | Qualitative | n=11 (mothers) | Help-seeking | Better awareness/understanding from practitioners, self-blame |
| Ulman & Straus (2003) | US | Quantitative | n=1,023 (children) | Characteristics | Gender (parent), age (child) |
| Walsh & Krienert (2009) | US | Quantitative | n=108,231 (incidents) | Characteristics | Age (child), gender (child), ethnicity (child), weapon |
| Walsh & Krienert (2007) | US | Quantitative | n=17,957 (young offenders) | Characteristics | Age (child), gender (child), gender (parent), physical CPVA, weapon |
| Williams, Tuffin & Niland (2017) | New Zealand | Qualitative | n=8 (mothers/grandmothers) | Help-seeking | Lack of practitioner understanding, parent-blame, self-blame |
| Zhang, Cai, Wang, Tao, Liu & Craig (2019) | China | Quantitative | n=1,134 (high school students) | Risk factors | Previous domestic violence/abuse, maternal rejection, family conflict, divorce |
| Zvara, Mills-Koonce & Cox (2016) | US | Mixed | n=204 (children) | Risk factors | Previous sexual abuse (parent), parenting style |
